# Genetic Structure of Earthworm Populations at a Regional Scale: Inferences from Mitochondrial and Microsatellite Molecular Markers in *Aporrectodea icterica* (Savigny 1826)

**DOI:** 10.1371/journal.pone.0101597

**Published:** 2014-07-08

**Authors:** Magally Torres-Leguizamon, Jérôme Mathieu, Thibaud Decaëns, Lise Dupont

**Affiliations:** 1 University Paris Est Créteil, Institute of ecology and environmental sciences of Paris, Créteil, France; 2 Unité de Zoologie Forestière UR0633, Institut National de la Recherche Agronomique, Orléans, France; 3 University Pierre and Marie Curie, Institute of ecology and environmental sciences of Paris, Paris, France; 4 EA 1293 ECODIV, University of Rouen, Mont Saint Aignan, France; Laboratoire de Biologie du Développement de Villefranche-sur-Mer, France

## Abstract

Despite the fundamental role that soil invertebrates (e.g. earthworms) play in soil ecosystems, the magnitude of their spatial genetic variation is still largely unknown and only a few studies have investigated the population genetic structure of these organisms. Here, we investigated the genetic structure of seven populations of a common endogeic earthworm (*Aporrectodea icterica*) sampled in northern France to explore how historical species range changes, microevolutionary processes and human activities interact in shaping genetic variation at a regional scale. Because combining markers with distinct modes of inheritance can provide extra, complementary information on gene flow, we compared the patterns of genetic structure revealed using nuclear (7 microsatellite loci) and mitochondrial markers (COI). Both types of markers indicated low genetic polymorphism compared to other earthworm species, a result that can be attributed to ancient bottlenecks, for instance due to species isolation in southern refugia during the ice ages with subsequent expansion toward northern Europe. Historical events can also be responsible for the existence of two divergent, but randomly interbreeding mitochondrial lineages within all study populations. In addition, the comparison of observed heterozygosity among microsatellite loci and heterozygosity expected under mutation-drift equilibrium suggested a recent decrease in effective size in some populations that could be due to contemporary events such as habitat fragmentation. The absence of relationship between geographic and genetic distances estimated from microsatellite allele frequency data also suggested that dispersal is haphazard and that human activities favour passive dispersal among geographically distant populations.

## Introduction

Earthworms represent one of the largest reservoirs of animal biomass and the main invertebrate group of soil ecosystem engineers in most terrestrial temperate ecosystems [1]. They play a key role in soil functioning: they relocate surface litter or organic matter throughout the soil profile [2], [3], affect microbial activity [4], and have a significant effect on organic matter mineralisation and soil biogeochemical cycles [5]. They modify soil structure *via* the construction of biogenic aggregates and galleries [6], resulting in differences in aeration and drainage [7]. Earthworms also influence plant growth [8] and plant community structure [9], [10], and can be used as indicators of habitat quality [11], [12] and as biomarkers in toxicity tests [13].

Despite their fundamental impact on soil ecosystems, the spatial population dynamics of earthworms is poorly understood. In particular, there is little information on the amount and spatial distribution of genetic variation in earthworm populations. Few studies have simultaneously investigated the influence of historical events, such as glacial periods, and contemporary processes, such habitat fragmentation, on the genetic diversity of these species. In their review on the genetic structure of soil invertebrate populations, Costa et al. [14] cite only seven studies of earthworm populations. They conclude that earthworm populations generally show a complicated pattern of gene flow, with a weak relationship between genetic and geographic distances. Population genetic structure of earthworms is therefore likely to be strongly influenced by human activities. In an agricultural landscape, the spatial distribution of earthworm species is expected to be fragmented, with patches of suitable habitat being separated by large areas of unsuitable habitat. Furthermore, it has been shown that earthworms are negatively affected by the intensity of agriculture [15] and in particular by the use of pesticides [16]. The consequences of landscape spatial structure on genetic diversity depend on the rate at which individuals move between patches of suitable habitat. In particular, restricted dispersers, such as earthworms, are likely to be prone to local extinction due to stochastic processes [17]. However, it has also been suggested that the rate of gene flow and the amount of genetic variation may actually increase as habitats become more fragmented [18]. More earthworm population genetics studies are needed to determine (i) how earthworms move between patches, (ii) how spatial structure affects the stability and dynamics of spatially structured earthworm populations, and (iii) how the landscape affects genetic diversity.

Our model earthworm species, *Aporrectodea icterica* is an abundant species commonly found in agricultural soils [19]. It belongs to the endogeic ecological type (i.e. species living and foraging in or immediately below the rhizosphere making horizontal burrows through the soil to move around and to feed) [20], although *A. icterica* is also believed to feed at least partly on leaf litter [21]. This diploid, obligatory bi-parental species [22] is native to the temperate zones of Europe [20], but is an invasive species in North America [23]. Its taxonomic status is firmly grounded and the species has distinct morphology making it easy to recognise. Its dispersal behaviour has been studied in laboratory [11], [24] and it has been used in ecotoxicological studies [19], [25], [26]. At the genetic level, a recent study of two *A. icterica* populations revealed the existence of two mitochondrial lineages with divergence values ranging from 10% to 11% [27]. Such highly divergent mitochondrial lineages have been reported in several other earthworm morphospecies (e.g. [28], [29], [30]). In *A. icterica*, nuclear analysis indicates that the two lineages interbreed [27], demonstrating that they belong to the same species.

Deep mitochondrial divergences within morphospecies can be attributed to population isolation within distinct periglacial refugia [29]. When the divergent lineages were found in sympatry, such as in *A. icterica*, it was suggested that lineages came into contact and mixed during recolonisation, during the warmer interglacial periods [29]. Given the low vagility of earthworms, we hypothesise that this mixing is in large part due to human activities which have accelerated the rate of organism dispersal, and brought previously allopatric species into contact [31]. During this secondary contact, weak reproductive barriers between lineages and fertile hybrids with little or no reduction in fitness can lead to genetic assimilation and loss of genetic distinctness between the hybridizing lineages, and the possible extinction of one or both parental lineages [31]. For recent or in progress hybridization events, introgressed mitochondrial and nuclear genes are predicted to display cytonuclear disequilibrium [32] (i.e. non-random association of alleles or genotypes at a nuclear locus with haplotypes of cytoplasmically inherited organellar DNA [33]).

Here, we focus on the genetic structure of seven earthworm populations sampled at a regional scale (<100 km^2^), comparing the spatial regional patterns obtained using mitochondrial (mtDNA) and nuclear (nDNA) molecular markers. We discuss the role of evolutionary forces including genetic drift and contemporary gene flow, large-scale landscape changes (e.g. glacial periods) and anthropogenic effects in structuring the genetic diversity and in the differentiation of populations.

## Materials and Methods

### Sampling and DNA extraction

**Figure 1:**
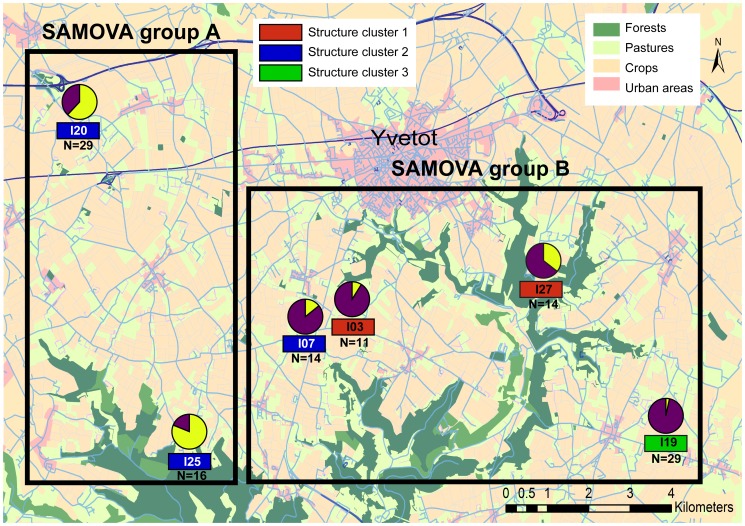
Geographical distribution of Yvetot populations and repartition of *cytochrome oxidase subunit I gene* (COI) lineages. Lineage 1 (L1) is shown in yellow and Lineage 2 (L2) is shown in purple. Groups revealed by the SAMOVA analysis of mitochondrial data and STRUCTURE analysis of microsatellite data are shown. Land use is also indicated.

In April 2010, 218 *A. icterica* individuals were collected from seven populations in Normandy (northern France). Six populations were located in >6 year-old pastures (on average, clay = 16%, silt = 64% and sand = 20%, mean pH: 6.1, C: 23 g.kg^−1^, N: 2.3 g.kg^−1^, C/N: 10), within a distance of 3 to 10 km from the city of Yvetot (I03, I07, I19, I20, I25 and I27). Each proprietor gave his agreement for sampling to J. Mathieu who should be contacted for future permissions. These pastures are grazed by dairy cattle from mid-March to mid-September with a stocking rate of 2–6 animal units ha^−1^ depending on the season, and spread with cow manure each year. Plant cover consisted mainly in *Festuca elator L.*, *Phleum pratense*, *Trifolium repens L.*, and *Lolium*. The seventh population was located 35 km away from Yvetot (IR), near the University of Rouen in a location for which no specific permission was required (Fig. 1 and Table 1 for GPS coordinates). This field study did not involve endangered or protected species.

**Table 1:** Genetic diversity in *A. icterica* populations.

				Mitochondrial data					Microsatellite data				
Locality	Code	Latitude (N)	Longitude (E)	*N*_mt_	*N*_h_	*r*_(11)_	*h*	*π*	*N*_ms_	*N*_all_	*A*_(22)_	*H*_e_	*F*_is_
**Yvetot**	**I03**	49.589	0.745	11	2	1.000	0.182	0.021	28	2.57	2.54	0.339	0.112
	**I07**	49.586	0.730	14	3	0.967	0.264	0.032	24	3.86	3.84	0.515	**0.386**
	**I19**	49.568	0.858	29	3	0.759	0.135	0.009	43	4.14	3.80	0.550	**0.379**
	**I20**	49.628	0.648	29	2	0.999	0.488	0.053	33	2.57	2.48	0.395	**0.176**
	**I25**	49.564	0.687	16	5	2.896	0.742	0.042	31	3.57	3.41	0.513	**0.232**
	**I27**	49.606	0.815	14	4	2.571	0.626	0.060	34	3.43	3.32	0.514	**0.372**
**Rouen**	**IR**	49.459	1.077	21	8	3.060	0.600	0.048	25	3.14	3.14	0.497	**0.442**
**Total**				134	15	-	0.563	0.041	218	5.71	**-**	0.561	**0.419**

Sample locations and codes are given, together with mitochondrial data: sample size (*N_mt_*), number of haplotypes (*N_h_*), haplotypic richness after rarefaction to a population size of 11 (*r*_(11)_), haplotype diversity (*h*) and nucleotide diversity (*π*), and microsatellite data: sample size (*N_ms_*), mean number of alleles (*N_all_*) allelic richness after rarefaction to a population size of 22 (*A_r_*), expected heterozygosity (*H_e_*), estimator of the inbreeding coefficient *F*_is_ (significant values are in bold).

Individuals were preserved in pure ethanol for DNA analysis. Total genomic DNA was extracted from a segment of the anterior end of the earthworm using the CTAB extraction protocol: digestion using proteinase K, followed by protein precipitation with CTAB, a chloroform∶isoamyl alcohol (24∶1) wash and DNA precipitation with sodium acetate (3 M) and ethanol.

### Mitochondrial DNA amplification and sequencing

A fragment of the *cytochrome c oxidase subunit I* mitochondrial gene (COI) was amplified and sequenced using the universal primers LCO1490 and HCO2198 [34]. For the I20 and IR populations, sequences were taken from Torres-Leguizamon et al. [27] (GenBank accession numbers JN381881–JN381930). Each amplification mixture (25 µl) contained 10 ng DNA, 12.5 µl of Taq PCR Master Mix (Qiagen, Hilden, Germany) and 0.25 µM of each primer. Polymerase chain reactions were performed using an initial denaturation step at 94°C for 3 min, followed by 40 cycles of the three following steps: denaturation at 94°C for 30 s, annealing at 49°C for 1 min and extension at 72°C for 1 min 30 s. The final extension was done at 72°C for 10 min. PCR products were purified using Microclean (Microzone Limited, Haywards Heath, UK). Both strands of amplicons were sequenced using Big-Dye Terminator Cycle sequencing kit version 1.1 (Applied Biosystems, Foster City, CA, USA) according to the standard protocol used in the genomic platform at the Mondor Institute of Biomedical Research (Créteil, France). Sequences were deposited in GenBank (Accession numbers: KF856627–KF856710).

### Mitochondrial DNA sequence analysis

COI sequences were aligned manually using BioEdit v. 7.0.5.3 [35]. For each population, haplotype diversity (*h*) and nucleotide diversity (*π*) were estimated using DNAsp v. 5.10 software [36]. Haplotypic richness after rarefaction to a population size of 11 individuals was estimated using Contrib software [37]. Departure from neutrality was tested using Fu's *Fs* [38] and Ramos-Onsins and Rozas' *R2* statistic [39], which are powerful tests for detecting recent population expansion under assumptions of neutrality. *R2* is appropriate for small sample sizes [39]. The significance of *R2* and *Fs* were evaluated by comparing their observed values with their null distribution, generated by 10 000 random replicates using the empirical population sample size and observed number of segregating sites implemented by DnaSP v. 5.10. [36].

To describe the phylogenetic relationships between haplotypes, a statistical parsimony network was constructed using TCS v. 1.21 [40]. The divergence among haplotypes was calculated in MEGA 5 using the mean uncorrected p-distance [41].

To investigate the regional structure of *A. icterica* populations at the mitochondrial level, the overall genetic differentiation between populations was first estimated by calculating the global Φ_st_ in Arlequin v. 3.5.1.2 software [42]. We then performed a spatial analysis of molecular variance using SAMOVA software [43]. Clusters are identified based on geographic proximity and genetic homogeneity [43]. The geographic coordinates of each sampled locality were used as spatial information. Simulations were conducted with ‘K’ (number of groups) ranging from two to seven and each simulation annealing process was repeated 100 times. The clustering giving the highest Φ_ct_ value, corresponding to the optimal number of groups, was selected.

### Microsatellite genotyping

Individuals were genotyped using seven microsatellite loci: Ai45, Ai56, Ai68, PB10D, 2PE40, 2PE70, C4 [27]. For individuals from the I20 and IR populations, genotypes were taken from Torres-Leguizamon et al. [27]. The seven loci were amplified by a touchdown PCR procedure that included an initial denaturation step of 3 min at 94°C, followed by 35 s at 94°C, 45 s at the initial temperature T_a_ +8°C, 10 cycles in which the temperature was decreased by 1°C per cycle, 1 min at 72°C, 25 cycles of 35 s at 94°C (except for PB10D and C4 for which 30 cycles were done), 45 s at Ta −2°C, 1 min at 72°C, with a final elongation step of 10 min 72°C. Each amplification mixture (15 µl) contained 10 ng/µl DNA, 1X reaction buffer (GoTaq Flexi buffer 5X), 2.5 mM of MgCl_2_ (except for Ai56 and Ai68 for which 1.5 mM was used), 0.5 µM dNTPs, 0.25 µM of each primer and 0.5 units of GoTaq Flexi DNA polymerase (Promega, Madison, WI, USA). PCR products were loaded on an ABI 310 sequencer along with the LIZ500 size standard; alleles were scored using Genescan software (all from Applied Biosystems, Foster City, CA, USA).

### Statistical analysis of microsatellite variation

In each population, the genetic diversity was analysed by computing allelic frequencies, number of alleles (*N*_all_) and unbiased expected heterozygosity (*H*_e_) using Genepop v. 4 software [44]. To take into account differences in sample size, allelic richness (A) after rarefaction to a population size of 22 individuals was estimated using FSTAT v. 2.9.3. software [45]. Exact tests for genotypic linkage disequilibria and deviations from Hardy-Weinberg equilibrium (HWE) were computed using Genepop v. 4 [44]. The sequential Bonferroni method was applied to adjust for multiple comparisons. Weir and Cockerham's (1984) estimator of the inbreeding coefficient *F*_is_ was calculated using Genepop v. 4 [44]. The presence of null alleles was tested using Micro-Checker v. 2.2.3 software, in which the Oosterhout method [46] was implemented and potential frequency of null alleles was estimated.

We tested for deviation from mutation-drift equilibrium in the study populations using the approach detailed in Cornuet & Luikart [47] and implemented in their software BOTTLENECK v. 1.2.02. Using a Wilcoxon test, observed heterozygosity was compared with the heterozygosity expected under equilibrium considering a two-phase mutation model (TPM) recommended for microsatellite data [48]. Recently founded populations are expected to show a transient excess of expected gene diversity, whereas expanding populations (e.g. recovering from a bottleneck) or populations resulting from immigration from differentiated sources should show a deficit in expected gene diversity [47].

To investigate the genetic structure among populations, a G-test of allelic differentiation was carried out using Genepop v. 4 [44]. In addition, we performed a principal component analysis (PCA) on gene frequency data using PCAGen v. 1.2.1 software (available at http://www2.unil.ch/popgen/softwares/pcagen.htm). The significance of each principal component was assessed from 1000 permutations. We also used the software STRUCTURE v. 2.3.1 [49] to estimate the number of genetic clusters (K) present among all populations. This software generates clusters of individuals based on the Hardy-Weinberg model of genotypic distribution. Simulations were run using the admixture model without prior population information. We modelled cluster assignments for K ranging from 1 to 10. We performed 25 independent runs for each K value to confirm consistency across runs. In all simulations, we applied a burn-in period of 10 000 iterations and 100 000 Markov chain Monte Carlo iterations. To determine the most likely value of K, we used the ΔK method [50].

To estimate recent migration rates and test for significant cases of assignment to populations other than the population of origin (i.e. first-generation dispersers) we used the Bayesian method [51] implemented in GeneClass2 v. 2.0. [52] paired with a Monte Carlo resampling method for computation of assignment probabilities for each population [53] using 10 000 simulated individuals.

To test for the null hypothesis of independence between genetic and geographic distances, the logarithm of Euclidian geographic distances were plotted against *F*_st_/(1−*F*_st_) to compute a linear relationship following the recommendations of Rousset [54] and Mantel tests [55] were performed using Genepop v. 3.4 [44] across 100 000 permutations.

### Cytonuclear disequilibrium analysis

Departures from random cytonuclear associations were tested using the CNDm programme [56]. The analyses were carried out by encoding mitochondrial haplotypes as two synthetic lineages (L1 and L2). Normalised cytonuclear disequilibria (CND) were calculated following Asmussen & Basten [33] for allelic associations, and significance levels were tested using Fisher's exact test.

## Results

### mtDNA genetic variation

The amplified COI fragment contained a homopolymer poly-C. In most of the reactions, the sequence became mixed after the poly-C, most probably because of polymerase stutter. Sequences were thus truncated (fragment length <200 bp). Consensus sequences shorter than 374 bp were excluded of the analysis. Over the whole mtDNA data set (134 sequences), we detected 15 haplotypes defined by 34 parsimony informative sites (9%) among 44 variable sites (12%). Within populations, haplotype diversity (*h*) ranged from 0.135 to 0.742 and nucleotide diversity (*π*) ranged from 0.00864 to 0.05973 (Table 1). Populations displaying low haplotypic richness were I03, I07, I19 and I20 (r_(11)_≤1) while I25, I27 and IR showed relatively high values of haplotypic richness (2.5 to 3). None of the Fu's *Fs* and *R2* values were significant.

**Figure 2:**
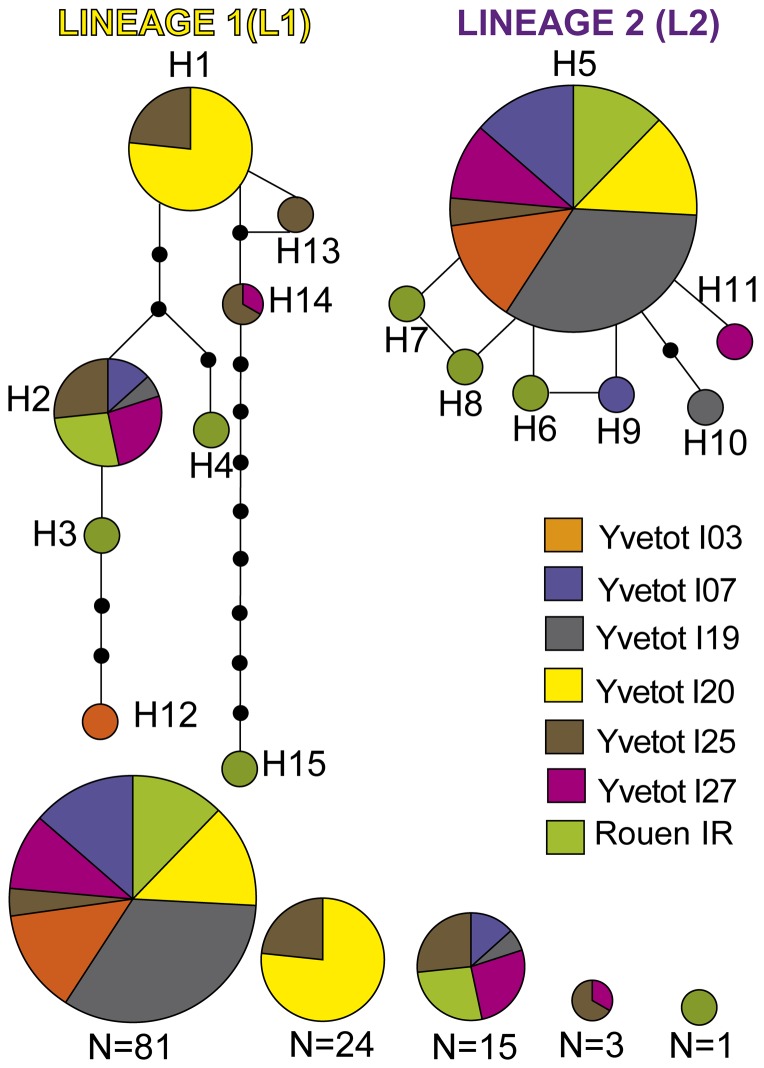
*Cytochrome oxidase subunit I* gene (COI) haplotype 95% statistical parsimony network for Yvetot and Rouen *A. icterica* samples. H1 and H5 represent presumed ancestral sequence. Circle size is relative to the proportion of each haplotype in the sample. Mutational steps are indicated by small black circles.

The haplotype distribution at the population level is shown in Figure 2. This haplotype network illustrates the relationships between the 15 haplotypes and shows a clear separation of *A. icterica* haplotypes into two divergent lineages L1 and L2 (Figs. 1 and 2). Both lineages showed a high percentage of divergence (8.7%). L1 consisted of 47 individuals and 8 haplotypes, two of which were abundant (H1 and H2). Within L1, haplotypes were more divergent (i.e. separated by several mutational steps) than within L2. L2 included sequences from 87 individuals and the most abundant haplotype (H5) was found in all populations, representing over half the L2 individuals (60.5%). The remaining seven L2 haplotypes were relatively infrequent but all closely related. They differed from the most common haplotype H5 by at most only two mutation steps.

Φ_st_ analysis showed significant genetic structure at the level of the whole study (7 populations, Φst = 0.324, p<0.001). In terms of regional mitochondrial structure, the SAMOVA showed that the highest significant value (Φ_ct_ = 0.409) was obtained when the populations were split into two groups (Fig. 1): group A corresponded to the I20 and I25 populations and group B was composed of the remaining populations in the Yvetot area (I03, I07, I27, I19) and the IR population (not shown in Fig. 1).

### nDNA genetic variation

Among the seven microsatellite loci, the number of alleles per locus ranged from one to seven (Table 2). None of the loci showed significant linkage disequilibrium. Genetic diversity indices varied among populations (Table 1), with the I03 and I20 populations showing lower values (A = 2.54, H_e_ = 0.339 and A = 2.48, H_e_ = 0.395, respectively) than the other populations (3.14<A<3.84 and 0.497<H_e_<0.515). Depending on the population, the Hardy-Weinberg expectations (HWE) test showed a significant deviation for some of the loci (Ai68, Ai56, C4, PB10D and 2PE70). However, null alleles were suspected for several loci (Table 2). The estimated frequencies of null alleles ranged from 12.1% (locus 2PE70, population I19) to 40.9% (locus C4, population I19). The data set was thus corrected for null alleles and both data sets (original and corrected) were used for analyses based on allelic frequencies.

**Table 2:** Hardy-Weinberg equilibrium P-value (significant values after sequential Bonferroni correction are in bold) together with the estimation of null allele frequency (in parentheses) for *A. icterica* microsatellite markers in each study populations.

	Locus ID (Number of alleles)						
Population	Ai45 (4)	Ai56 (7)	Ai68 (8)	PB10D (4)	2PE40 (3)	2PE70 (3)	C4 (11)
**I03**	0.296	1.000	1.000	0.077	ML	0.442	0.093 (0.155)
**I07**	0.500	0.609	0.067 (0.146)	**0.001** (0.256)	ML	**0.001**	**0.000** (0.385)
**I19**	0.116	0.459	0.002 (0.216)	0.330	0.144	0.013 (0.121)	**0.000** (0.409)
**I20**	0.105	ML	0.755	0.935	ML	**0.001** (0.274)	**0.000** (0.252)
**I25**	0.031	0.405	0.463	0.110	ML	0.002 (0.257)	**0.000** (0.314)
**I27**	0.613	0.217	**0.000** (0.182)	**0.000** (0.210)	ML	0.163	**0.000** (0.346)
**IR**	0.315	**0.000** (0.318)	0.002 (0.230)	1.000	1.000	0.002 (0.284)	**0.000** (0.306)

ML: monomorphic locus.

When testing for mutation-drift equilibrium, a significant gene diversity excess was detected only in the I27 population using the original dataset, but also in the I07, I20, I25 and IR populations using the corrected dataset without null alleles, suggesting that these populations are good candidates for recent demographic disequilibrium arising from a population bottleneck.

**Figure 3:**
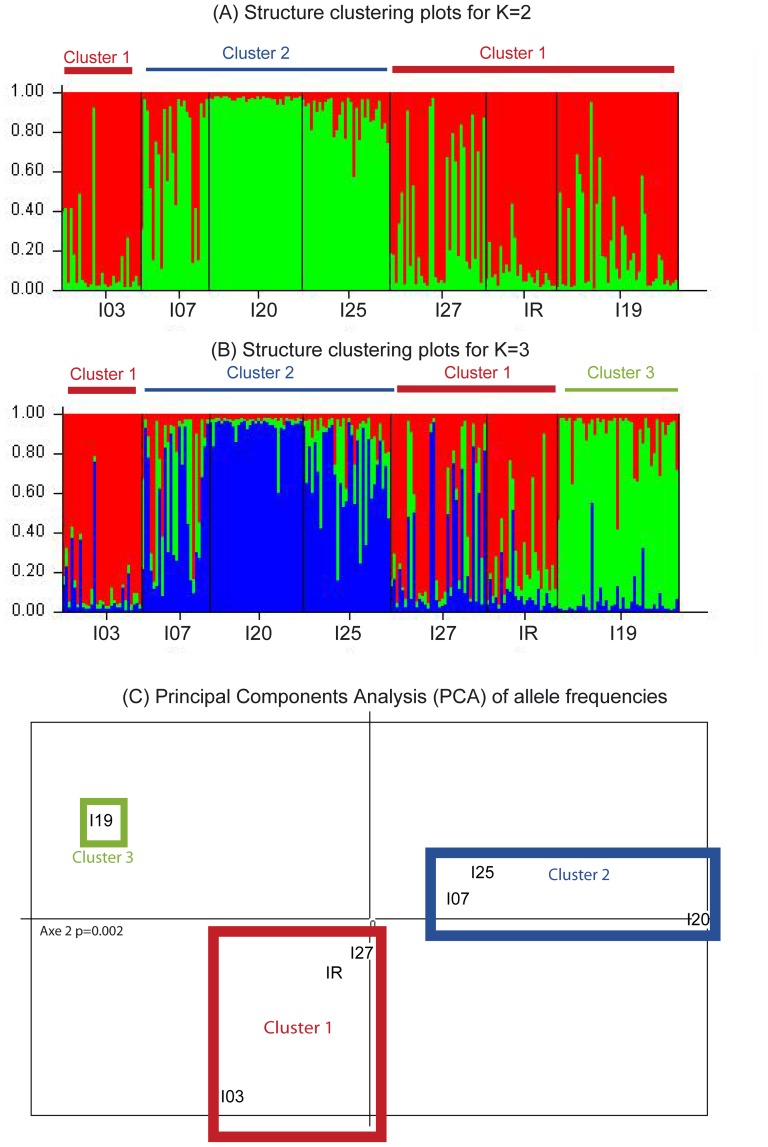
Genetic clustering of *A. icterica* populations based on analysis of microsatellite data. A and B. STRUCTURE Q plots representing the number of genetic nuclear groups for K = 2 and K = 3 respectively in *A. icterica* for I03, I07, I20, I25, I27 and IR populations. Each individual is represented by a vertical bar showing degree of admixture. C. Principal components analysis (PCA) of microsatellites allele frequencies for the whole dataset. Level of significance was derived from 1000 permutations and significant P-value is shown.

Significant genetic structure was revealed at the level of the whole study (G-test, p<0.001). No pattern of isolation by distance was observed among the six Yvetot populations (p = 0.342 and p = 402 using the original and the corrected data set respectively). Clustering analysis (Figs. 3A and 3B) clearly indicated genetic similarities among the I03, I27 and IR populations (Cluster 1) and among I07, I20, I25 populations (Cluster 2). The case of I19 was more ambiguous. The highest ΔK value was obtained for K = 2 (ΔK = 60.90, Fig. 3A), although the ΔK value for K = 3 was comparable (ΔK = 47.87, Fig. 3B). For K = 2, the I19 population was grouped with Cluster 1, whereas for K = 3, it formed a third group. The results of the PCA on allelic frequencies were in agreement with the results of the clustering analysis (Fig. 3C). The populations were separated into two major groups along the second axis of the PCA, which was highly significant (original dataset, p = 0.002 and corrected data set, p = 0.001). Population I19 was genetically isolated from the two clusters. Within each group, I03 and I20 populations were highly differentiated from the other two populations in their respective group (Fig. 3B).

Contemporary gene flow was detected at this regional scale with 64 individuals (29%) identified as first-generation dispersers. Among these 64 individuals, 30 belonged to Cluster 1, 25 belonged to Cluster 2 and the 9 remaining migrants belonged to the I19 population (Table 3). Most of the first-generation dispersers were assigned to populations belonging to the same cluster (19 first-generation dispersers from Cluster 1 out of 30 and 18 first-generation dispersers from Cluster 2 out of 25). Populations I03, I20 and I19 showed the lowest number of dispersers (5, 6, and 3 migrants, respectively).

**Table 3:** Inference of gene flow between populations belonging to each group defined using Structure: the percentage of individuals assigned to each locality as estimated by GeneClass2 is presented.

	Cluster 1			Cluster 2			Cluster 3
	I03	I27	IR	I07	I20	I25	I19
I03	82	3	12	0	0	0	0
I27	7	68	36	25	3	6	2
IR	7	6	44	0	0	0	2
I07	0	15	0	42	3	16	2
I20	0	0	0	8	82	6	0
I25	0	3	0	21	9	65	0
I19	4	6	8	4	3	6	93

Source localities are given in rows, recipient localities in columns.

### Relationships between mitochondrial lineages and microsatellite alleles

The test for overall non-random association between microsatellite alleles and mitochondrial lineages revealed significant cytonuclear disequilibrium after Bonferroni correction for four of the most polymorphic microsatellite loci (Table 4). Three alleles showed significant association with L1, whereas 5 alleles were significantly associated with L2.

**Table 4:** Cytonuclear linkage disequilibrium between *A. icterica* mitochondrial lineages and microsatellite alleles, estimated using CNDm software (Basten & Asmussen 1997).

Microsatellite locus (Number of alleles*)	mtDNA lineage	
	L1	L2
Ai45 (3)	-	-
Ai56 (5)	129	133
Ai68 (8)	-	118
PB10D (4)	220	178, 182
2PE40 (3)	-	-
2PE70 (3)	-	-
C4 (9)	176	178

Alleles significantly associated with mitochondrial lineage, after Bonferroni correction, are indicated.

* the analysis was only executed for samples for which both COI haplotypes and multilocus microsatellite genotypes were scored.

The test was also carried out within each population that showed both lineages, with the rarest representing at least 30% (IR, I20 and I27). The association between allele 129 at the Ai56 locus and L1 was suggested in the IR population, but was not significant after Bonferroni correction (p = 0.037). In addition, there was a trend for an association between allele 178 of the PB10 locus and L2 (p = 0.092) in the I20 population.

## Discussion

### Low genetic diversity within A. icterica populations

The level of polymorphism detected in *A. icterica* populations using microsatellite markers and COI sequences was surprisingly low (Table 5 and Table 6). The seven microsatellite markers showed low genetic variability with only 3 to 11 alleles over all loci. This polymorphism was lower than that reported in all other microsatellite datasets on earthworm populations (Table 5). For instance, the mean number of alleles per locus (N_A_) ranged from 2.57 to 4.14, but values of 5 to 17 alleles have been reported in other earthworm species (*Eisenia fetida* [57] and *Lumbricus terrestris* [58], respectively). Similarly, the sequenced fragment of the COI gene (374 bp) displayed low genetic variability in comparison to other earthworm species, despite the relatively restricted geographical scale and the short length of sequenced fragment tested in this study. For instance, only 12% of sites were polymorphic, but 33% (*Hormogaster elisae* [30]) to 36.5% (*Metaphire sieboldi* [59]) of sites are polymorphic in other earthworm species.

**Table 5:** Polymorphism of microsatellite loci in earthworm species.

Morphospecies	Sampling design	Geographical range	*N*_ind_	*N*_loci_	*N*_A_	*H*_e_	Reference
*Allolobophora chlorotica*	2 populations in 2 countries	∼500 km	62	8	6.63–9.63	0.725–0.774	[63]
*Aporrectodea icterica*	7 populations in northern France	<100 km^2^	218	7	2.57–4.14	0.339–0.550	This study
*Aporrectodea longa*	1 site	<0.50 km^2^	31	11	7.18	0.654	[73]
*Eisenia fetida*	3 vermiculture stocks	NA	70	16	5.00–5.75	0.630–0.660	[57]
*Hormogaster elisae*	1 site	<0.50 km^2^	26	10	12.5	0.821	[74]
	1 site	0.064 km^2^	75	4	7.32	0.890	[75]
*Lumbricus rubellus*	1 site	<0.50 km^2^	34	8	9.75	0.669	[76]
*Lumbricus terrestris*	1 site	<0.50 km^2^	32	10	12.8	0.853	[77]
	1 site	<0.50 km^2^	281	3	17	0.817	[58]

For each earthworm species for which microsatellites have been used for population genetics study, the number of polymorphic loci (*N*_loci_), mean number of alleles per locus (*N*_A_), and multilocus gene diversity (*H*_e_) are indicated.

NA: information not provided in the study.

**Table 6:** COI sequence polymorphism in earthworm morphospecies.

Morphospecies	Number of locations	Sampling area	Geographical range	N_ind_	*L*_seq_	*N*_h_	*P*_S_ (%)	*N*_L_	Reference
*Allolobophora chlorotica*	38	5 European countries	∼430 000 km^2^	153	582	54	NA	7	[63]
*Aporrectodea icterica*	7	Northern France	<100 km^2^	134	374	15	12	2	This study
*Aporrectodea trapezoides*	47	11 countries	∼5 510 000 km^2^	178	456	37	34	2	[78]
*Dendrobaena octaedra*	6	Southern Finland	∼52 000 km^2^	118	441	24	NA	1	[79]
*Hormogaster elisae*	7	Central Iberian Peninsula	<100 km^2^	82	658	38	33	6	[30]
*Metaphire sieboldi*	64	Southern Japan	∼300 000 km^2^	71	690	NA	36.5	1	[59]
*Rhinodrilus alatus*	21	Southeastern Brazil	∼16 000 km^2^	69	593	59	34	6	[80]

For each earthworm morphospecies for which COI has been recently used (since 2009) in a population genetics study, the length of sequence alignment (*L*_seq_), the number of haplotypes (*N*_h_), proportion of variable sites (*P*_S_) and the number of divergent lineages (*N*_L_) are indicated.

NA: information not provided in the study.

Ancient bottleneck events due to population isolation in periglacial refugia may be partly responsible for the current low genetic variation in this earthworm species. Among contemporary events, there are two major explanations for the low level of polymorphism in *A. icterica*: the occurrence of recent population bottlenecks and/or recurrent inbreeding due to reproduction between relatives. High inbreeding due to preferential mating among relatives (see for instance [60]) is unlikely since deviation from HWE was inconsistent across loci and populations and could be attributed to null alleles. In some *A. icterica* populations, inbreeding may nevertheless occur due to a decline in effective population size. Our results indeed suggested that some populations were recovering from a recent population bottleneck. Bottlenecks can occur following colonisation events because the number of initial colonists is often small and genetic drift may result in reduced genetic variation in a newly established population [17]. However, in an outcrossing species such as *A. icterica*, the likelihood of a genetic bottleneck is low because even only a few immigrants can introduce a large increase in genetic variation [61]. Only successive and drastic bottlenecks could have severely affected the genetic variation of *A. icterica*. Agricultural practices such as crop rotation can contribute to a fragmentation of the species habitat and thereby cause successive genetic bottlenecks [17]. In addition, geographic isolation of populations due to natural and artificial barriers to gene flow can accentuate the loss of genetic variability through genetic drift. Low levels of gene flow in fragmented habitats can even lead to extinction of local populations due to stochastic processes. Extinction-recolonisation is a classic metapopulation scenario, with periodic extinction of all individuals in a particular patch and subsequent recolonisation of this patch from surrounding areas [17].

### Relationship between genetic and geographic distances at a regional scale

A relatively high level of genetic differentiation was revealed among localities, regardless of the marker used. Interestingly, no relationship between genetic and linear geographic distances was observed at this regional scale (populations separated by less than 13 km in the Yvetot area), corroborating other earthworm population genetics studies (reviewed in [14], but see [30]). The lack of relationship between genetic differentiation and geographic distances was confirmed by the cluster analyses. At the nuclear level, populations were clustered into two major groups (Cluster 1 = I03, I27 and IR and Cluster 2 = I07, I20, I25), within which most of the first-generation dispersers were detected. It is noteworthy that two geographically close populations (I03 and I07) did not belong to the same cluster. There are two hypotheses that can explain the lack of correlation between the genetic and geographic distances. First, stochastic events, such as environmental changes, demographic factors (i.e. chance differences among individuals in survival or fecundity) and genetic drift may be more important than active dispersal in partitioning genetic variation at this scale (i.e. 1 to 15 km). Among earthworms, which are believed to be able to actively disperse at distances ranging from 4 to 14 m year^−1^ (review in [11], [62]), *A. icterica* is considered to be relatively vagile, being able to travel up to 500 m year^−1^ under conditions that trigger dispersal [11], [24]. Tracing active dispersal events requires a study at a finer scale (<500 m^2^).

Second, passive dispersal due to rain, floods, streams, birds, cattle or various human activities [62] may promote gene flow between geographically distant populations. In agricultural regions, such as in the Yvetot area, earthworms or cocoons are likely to be passively dispersed via various human activities that involve transporting soil or plant material, for instance (see [62] for review).

### Discordant patterns of mitochondrial and microsatellite genetic structure

Two divergent (8.7%) mitochondrial lineages were observed within studied populations of *A. icterica*. In the Yvetot area, most of the individuals from the I20 and I25 populations belonged to L1 whereas the majority of samples from I03, I07, I19 and I27 belonged to L2 (Fig. 1). In Rouen, the population was predominantly composed of individuals belonging to L2. Genetic differentiation was confirmed in the SAMOVA analysis, with a grouping along the same lines.

Divergent sympatric mitochondrial lineages often reveal the existence of cryptic species, particularly when divergence is confirmed in the nuclear compartment of the genome and/or when reproductive isolation between lineages has been demonstrated [29], [63]. In *A. icterica*, our results indicate that the two divergent lineages were randomly interbreeding with respect to mtDNA haplotypes over a relatively restricted geographical area. Deep mtDNA divergence despite clear interbreeding can reflect long periods of geographical isolation followed by secondary contact favouring gene flow, homogenising the nuclear genome over time.

Glaciation, which became increasingly severe throughout the Pleistocene, is known to have drastically modified species distributions [64]. Most organisms presently distributed across Europe retreated to refugia during glaciation ca. 18 000 years BP, mostly in the peninsulas of Iberia, southern France, Italy and the Balkans, and, in some cases, near the Caucasus and the Caspian Sea [64]. Although no common phylogeographic histories across Europe have been proposed, Taberlet et al. [65] highlighted some concordance in two postglacial colonisation routes: (1) from Iberia and southern France towards Scandinavia and (2) from a Balkan refugium towards south-eastern France. Recent analyses of earthworm communities have shown that past climate changes have left a deep footprint on present-day earthworm diversity patterns, from community to macroecological scales [66]. It appears that earthworms recolonised France from two large refugia in southern France. However, there has been also recolonisation from eastern Europe and north-eastern France, and two micro-refugia were probably conserved in very specific locations in the Vosges (north-eastern France) and in Brittany (north-west of France). However, these recolonisation sources are difficult to assess [66]. Nevertheless, we can assume that the divergent *A. icterica* lineages originated from distinct refugia and that they merged during post-glaciation recolonisation. MtDNA divergence may thus be the result of neutral differences within the species, representing a historical mark of divergent lineages that have remerged [67], [68]. Over time, haplotypes are lost due to genetic drift (i.e. lineage sorting), resulting in populations monophyletic for a single gene lineage [69]. Deep mtDNA divergence can only be maintained in a panmictic population with large effective population size, which effectively slows lineage sorting [68]. However, we argue that *A. icterica* has small effective population size and has undergone serial population bottlenecks during the process of post-Pleistocene recolonisation in northern Europe, further accentuated by recent bottlenecks due to habitat fragmentation. We therefore assume that *A. icterica* lineages have come into contact too recently for lineage sorting to be completed. Furthermore, human activities could be, at least in part, responsible for the merging of two divergent lineages. For instance, the contribution of historical human activities to the current pattern of spatial genetic structure was documented for numerous plant species (e.g. [70], [71], [72]). Overall, our results suggest both past and ongoing anthropogenic impacts on *A. icterica* population genetic structure.

Here, we investigated whether the process of remerging can be traced back by studying the cytonuclear disequilibrium within contemporary populations of *A. icterica*. In the global dataset, some nuclear alleles were non-randomly associated with one of the two mitochondrial lineages. Because of the low number of populations displaying enough copies of both lineages and because of the relatively low number of individuals for which both COI haplotypes and microsatellites genotypes were scored, this non-random association could not be confirmed at the population level. Thus we cannot completely exclude the possibility that the observed cytonuclear disequilibrium is due to genetic structuring at the scale of the study.

## Conclusions

Overall, this study confirmed general patterns of genetic variation observed in earthworms, such as (i) the importance of historical events for explaining their current genetic variation and (ii) the weak relationship between genetic and geographic distances suggesting the importance of passive dispersal in structuring earthworm populations. Nevertheless, some uncertainties persist such as the underlying cause of the mito-nuclear discordance and the respective roles of active *versus* passive dispersal in partitioning population genetic structure. In particular, it is critical to investigate how individual dispersal interacts with landscape structure. Further study is now needed to examine the extent to which barriers to movement and corridors that facilitate dispersal determine earthworm population connectivity in heterogeneous landscapes.
